# Improvement on Fully Filled Through Silicon Vias by Optimized Sputtering and Electroplating Conditions

**DOI:** 10.3390/ma12223713

**Published:** 2019-11-11

**Authors:** Fei Zhao

**Affiliations:** The 54th Research Institute of China Electronics Technology Group Corporation, Shijiazhuang 050081, China; fly.zf@163.com

**Keywords:** TSVs, sputtering, electroplating, interface effect

## Abstract

The high reliability of electroplating through silicon vias (TSVs) is an attractive hotspot in the application of high-density integrated circuit packaging. In this paper, improvements for fully filled TSVs by optimizing sputtering and electroplating conditions were introduced. Particular attention was paid to the samples with different seed layer structures. These samples were fabricated by different sputtering and treatment approaches, and accompanied with various electroplating profile adjustments. The images were observed and characterized by X-ray equipment and a scanning electron microscope (SEM). The results show that optimized sputtering and electroplating conditions can help improve the quality of TSVs, which could be interpreted as the interface effect of the TSV structure.

## 1. Introduction

With the increasing tendency for the application of miniaturization and high-speed communication, three-dimensional (3D) integrated circuits with through silicon vias (TSVs) have become promising candidates for building modules and systems of high speed, frequency, and density [1,2]. In order to enhance the quantity of transistors in the chips, the vertical stacking of TSVs plays an important role in the field of semiconductor devices, which broke through the bottleneck of traditional two-dimensional integration. By means of three-dimensional integration, TSV technology has been widely studied in recent years [3].

Zhang et al. illustrated the whole design and fabrication process of TSVs [4], wherein via filling is a crucial procedure which influences the resistivity and capacity of the electrical parameters, including the reliability of the whole circuit [5]. Until now, Cu has been considered to be the best TSV filling material because of its ultra-low resistivity and cost [6]. Achieving a robust and void-free Cu filling with excellent electrical conduction is the foundation of three-dimensional integration and the main research direction. As previously reported, ordinary electroplating cannot achieve a satisfactory effect on full filling due to the so high aspect ratio of TSVs [7]. Electroplating for vias was operated with difficulties, such as bonding the TSV wafer to an auxiliary wafer with seed layers [8], pretreating the wafers [9], adjusting the additives in the solution [10], using pulse-reverse current electrodeposition [11], and optimizing the models of simulation [12]. To optimize quality, additives including polyethylene glycol, bis-(3-sodiumsulfopropyl disulfide), and Janus Green B—used as suppressors, accelerators, and levelers, respectively—have been studied. Also, different current conditions, such as pulse, pulse-reverse, and periodic pulse-reverse, were employed [13]. The preconditions for copper deposition with a uniform microstructure have been studied and the type of gelatin additive has been reported [14]. In order to achieve 100% step coverage, a low leakage current, and also increase the growth rate, a thermal oxide/plasma enhanced chemical vapor deposition (PECVD) Tetraethylorthosilicate(TEOS) bilayer was formed [15]. In order to increase the manufacturing efficiency, a dry film photoresist was introduced in the through via filling process [16]. When developing a high quality TSV electroplating process, it is crucial to solve any problems relating to the parameters of the seed layers, solution, equipment and so on. However, only a few experimental results [15,16,17,18,19,20,21] have been reported on the effect of the parameters of seed layer sputtering and electroplating profiles on the quality of the filled copper, which has led to difficulty in optimizing the filling percentage in vias. For seed layer preparation, Song et al. [15] studied the novel deposition with an atomic layer deposition and electroless seed deposition, which showed significant potential for the higher aspect ratio of TSVs. ToF-SIMS 3D analyses of thin films have been carried out to study the uniformity of silicon dopant concentration in atomic layer thin films [17]. For plating conditions, a novel seedless TSV process using electrically conductive adhesives to cure silver nanowires has been studied in room temperature. This showed excellent conductivity due to the benefits of both poly(methyl methacrylate) and silver nanowires [18]. During plating, the relationship between the experimental variables, including current density, additive concentration, and different shapes of TSVs was systematically analyzed. This was assisted with pulsed power as the experimental power source and additive concentrations as the electroplating solution [19]. Analyses of the process, including cyclic voltammetry (CV) and in situ molecular resolution scanning tunneling microscopy (STM) techniques, have been utilized to illustrate the electroplating procedure [20,21]. It can be summarized that the above mentioned improvements rely mainly on the advanced equipment being complicated to operate.

In this paper, different sputtering approaches of the seed layers were employed to prepare the TSV samples, accompanied by the adjustment of various electroplating profiles. In order to investigate the improvement of the optimized sputtering and electroplating conditions on the quality of the TSVs, X-ray and scanning electron microscope (SEM) images were observed to characterize the structures of the samples. Furthermore, the mechanism of the optimized behavior was illustrated according to the interface effect of the TSV structure.

## 2. Materials and Methods 

**Figure 1:**
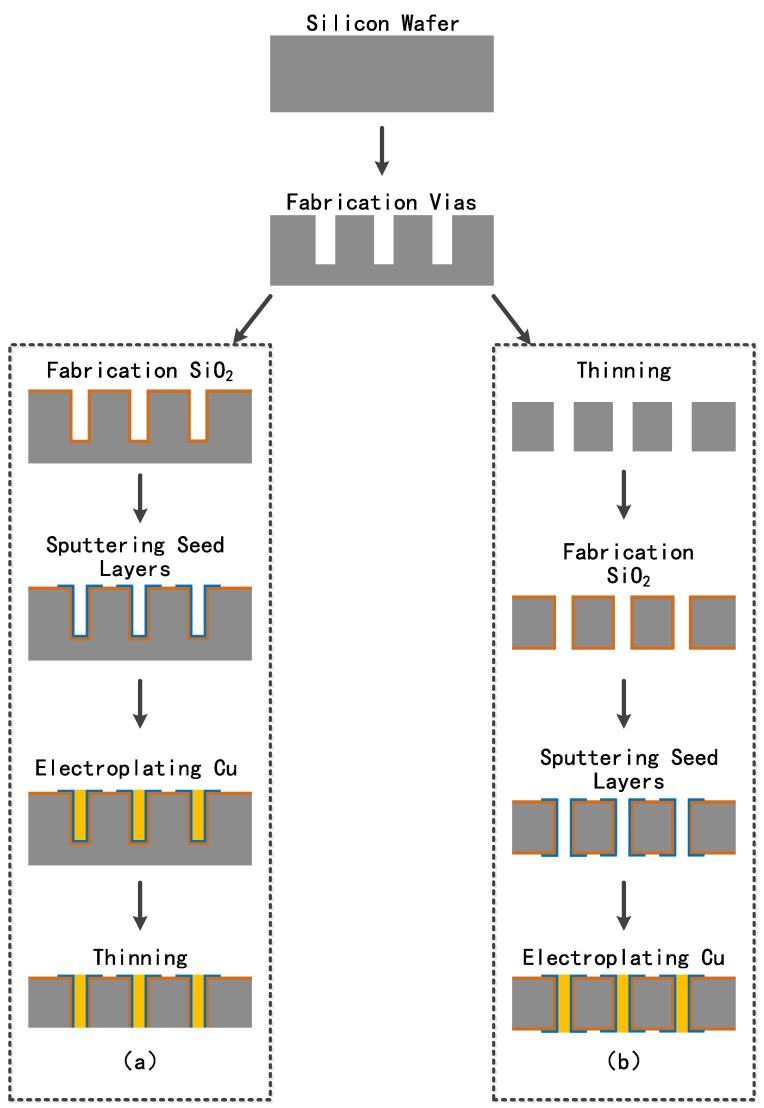
Illustration of through silicone via (TSV) fabrication process showing (**a**) blind vias and (**b**) through vias.

Four-inch silicon wafers with high resistivity of 4000 Ω•cm were used to fabricate vias with aspect ratios of 1:1 and 1:4 by the Bosch etching method. Two fabrication process flows comprising blind via filling and through via filling are presented in detail in Figure 1a,b.

The blind via filling process flow can be described as follows:

(a) The silicon wafer with blind vias was placed into the ICP-CVD equipment at 75 °C for 1 h to grow an SiO_2_ layer. Low-temperature growth of the SiO_2_ layer can prevent the damage caused by high-temperature thermal oxidation;

(b) The substrate with the SiO_2_ layer was then deposited with metal seed layers composed of Ti and Cu by magnetic controlled sputtering, where deposition power was the RF source; 

(c) After fabricating the seed layers, pretreatment was performed by various means before electroplating. Then, the silicon wafer was transferred into the plating solution quickly to develop the Cu pillar for filling the blind vias;

(d) The electroplated wafer was bonded to the glass wafer and thinned on the back side until the filled Cu was exposed. Then, the electroplated wafer was debonded and thinned on the top side in order to obtain a smooth surface for further process.

The through via filling process flow can be described as follows:

(a) Firstly, the silicon wafer with the blind vias was thinned on the back side to expose the through via; 

(b) Then, the silicon wafer with through vias was arranged to grow a SiO_2_ layer by ICP-CVD process;

(c) Magnetic controlled sputtering was carried out to deposit the metal seed layers composed of Ti and Cu; 

(d) The silicon wafer with seed layers was Cu-electroplated and rinsed into the electroplating solution.

**Figure 2:**
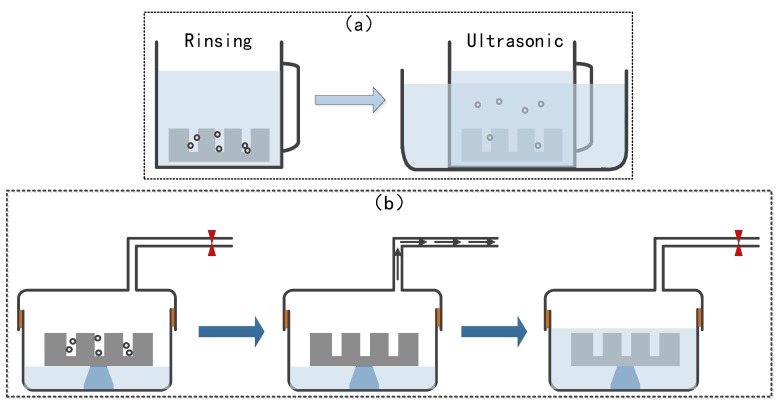
Illustration of pretreatment approaches before plating. (**a**) Rinsing in deionized (DI) water followed by an ultrasonic wave; (**b**) Vacuuming in a sealed environment followed by rinsing in DI water.

Compared with through via filling, blind via filling is more complicated and should be pretreated before being electroplated in order to prevent bubbles adhering to the sidewalls of the vias. Thus, the vias can be filled better to achieve a higher quality. The effective pretreatment methods are utilized in the following. As shown in Figure 2a, by rinsing the silicon wafers with blind vias into deionized (DI) water for different periods of time, the bubbles could be expelled to different extents, leading to improvement of the filling state, during which an ultrasonic wave helped to better eliminate the bubbles [3]. Figure 2b shows another effective approach for pretreating the wafers before electroplating, where wafers are placed into a container with a valve and then vacuumed to remove the bubbles hiding in the blind vias [7]. Then, DI water was injected into the container to maintain the condition of the treatment. In this paper, the process of through vias was utilized to study the improvement in filling quality.

## 3. Results and Discussions

### 3.1. Improvement of Sputtering

The effect of seed layer sputtering was studied and the improvements of the sputtering processes before Cu electroplating were illustrated.

**Figure 3:**
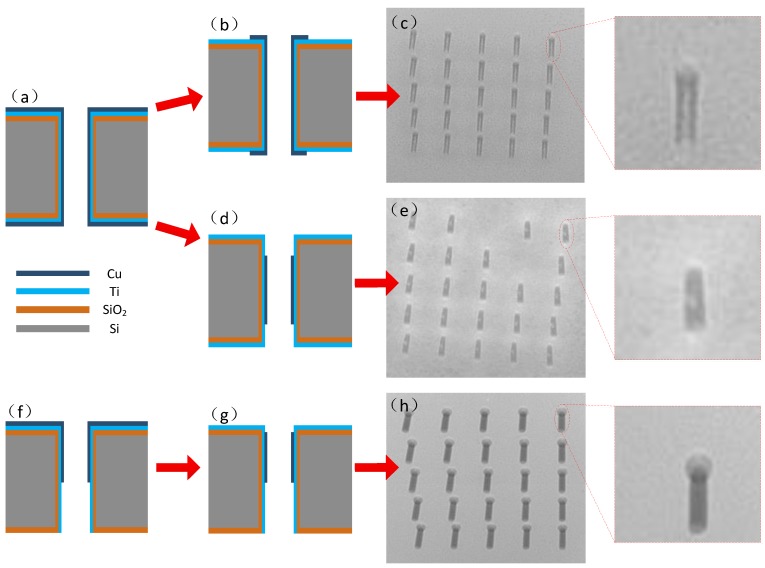
Schematic illustration and X-ray images of seed layer sputtering effect. (**a**) Double-side sputtering of TSV; (**b**) Double-side Cu-etched TSV with lithography; (**c**) X-ray image of electroplated sample (**b**); (**d**) Double-side Cu-etched TSV without lithography; (**e**) X-ray image of electroplated sample (**d**); (**f**) Single-side sputtering of TSV; (**g**) Single-side Cu-etched TSV without lithography; (**h**) X-ray image of electroplated sample (**g**).

Figure 3a presents the normal seed layer structure after sputtering. Magnetic controlled sputtering of metal layers including Ti and Cu was performed with an RF power of 200~400 W and Ar flow of 50~100 sccm for 10 min to 1 h to make sure that the sidewalls of the vias were fully covered with metal layers. After that, the silicon wafer with metal layers was lithographed both on the top and bottom side of the through vias, followed by Cu etching and photoresist striping resulting in Cu exposure only in the vias, shown in Figure 3b. By adjusting the additives and current density during the plating, the vias were electroplated. It can be seen in Figure 3c and the accompanying amplified image that gaps were hidden in the vias, revealing the unsatisfactory result of the filling. The defects of the filling can be attributed to either the residual photoresist during the lithography process or the mechanism of Cu growth.

In order to identify the key factor for the aforementioned reasons, another experiment was carried out. After the double-side sputtering of the Ti/Cu layers shown in Figure 3a, the lithography process was cancelled and Cu etching was performed directly, shown in Figure 3d. A significant improvement was evident, as seen in the X-ray images captured in Figure 3e, although voids still existed in the Cu pillars, as shown in the accompanying amplified image. According to the results, the mechanism of Cu growth was the more essential compared with the residual photoresist.

An advanced improvement of sputtering was carried out by top-side sputtering of the Ti/Cu layers, where Ti and Cu were the first and second layers, respectively. After that, Cu was etched leaving only one-third of Cu coverage closing the top side of the wafer surface, as shown in Figure 3f,g. It can be seen that, after electroplating, the through vias were fully filled without voids, illustrated in Figure 3h, proving again that the quality of the via filling is greatly affected by sputtering and its treatment.

**Figure 4:**
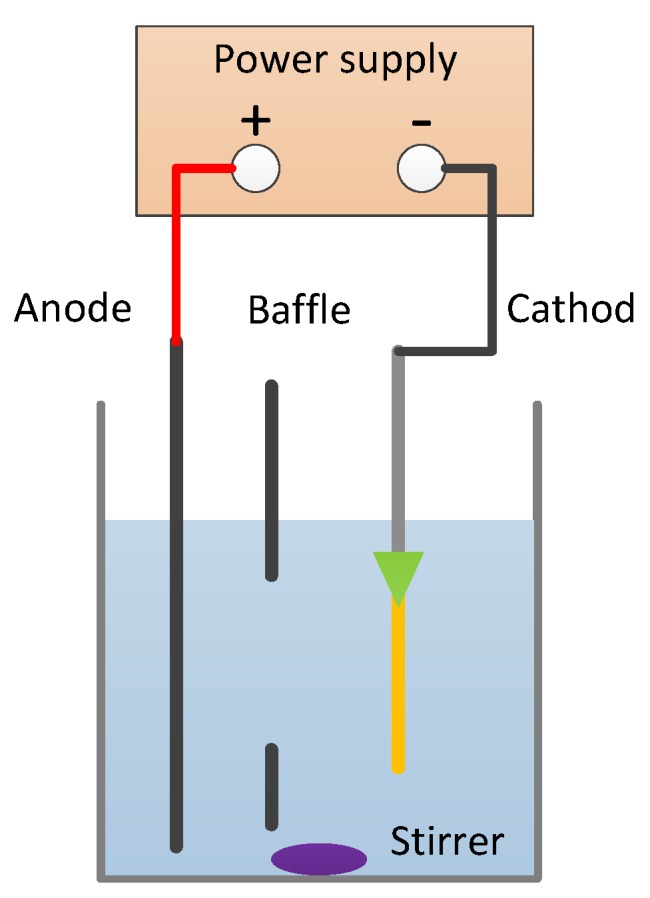
Schematic illustration of the electroplating solution with the anode of Cu plate and the cathode of the silicon wafer.

**Figure 5:**
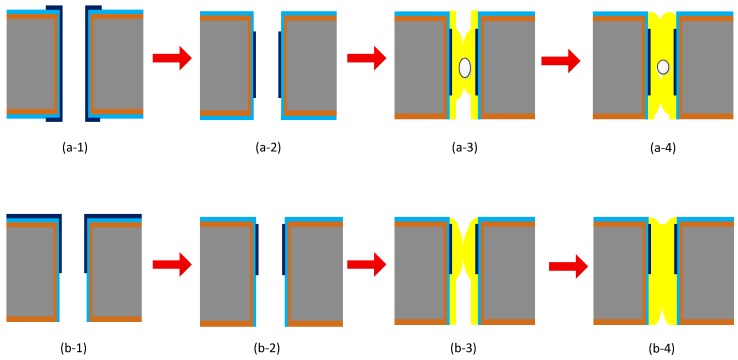
Schematic illustration of the mechanism for Cu growth. (**a**) Double-side sputtering of TSV; (**b**) Single-side sputtering of TSV.

Based on the experimental results, the mechanism of the above treatment was illustrated. As shown in Figure 4, electroplating occurs in the solution with the anode of Cu plate and the cathode of the silicon wafer. Cu particles are electrodeposited on the silicon wafer with metal layers including both adhesive and seed layers. Normally, Ti is an outstanding adhesive layer while Cu is the commonly used conductive layer in the circuit. At room temperature, the resistivity of Cu is 1.72 × 10^−8^ Ω·m, while the resistivity of Ti is 4.2 × 10^−8^ Ω·m. Ti has higher resistivity than Cu, indicating that it is much harder to electroplate a metal layer on Ti than Cu. In this case, an optimized structure of the metal layers could help to adjust the plating rate, thereby controlling the filling quality. As shown in Figure 5a-3, the vias made by double-side sputtering had more Cu than Ti in the through vias since the electroplating rate was fast at the opening, leading to voids. However, in Figure 5b-3, vias made by single-side sputtering had less Cu than Ti in the through vias since the electroplating rate was slower at the opening and did not produce voids. By increasing the electroplating time, the vias could be fully fed to obtain the ideal status.

### 3.2. Improvement of Electroplating Condition

The improvement of the electroplating condition was also studied during the process. Two groups of samples (40 µm in diameter/160 µm in depth; 150 µm in diameter/130 µm in depth) were fabricated and electroplated with three different stepwise current densities. The current densities during electroplating on the state of via filling were stepwise set. The volumes of the additives, including accelerator, stabilizer, and leveler, were 3, 5, and 10 mL, respectively. The plating process was assisted with a magnetic stirrer at a rate of 500~600 rpm. 

**Figure 6:**
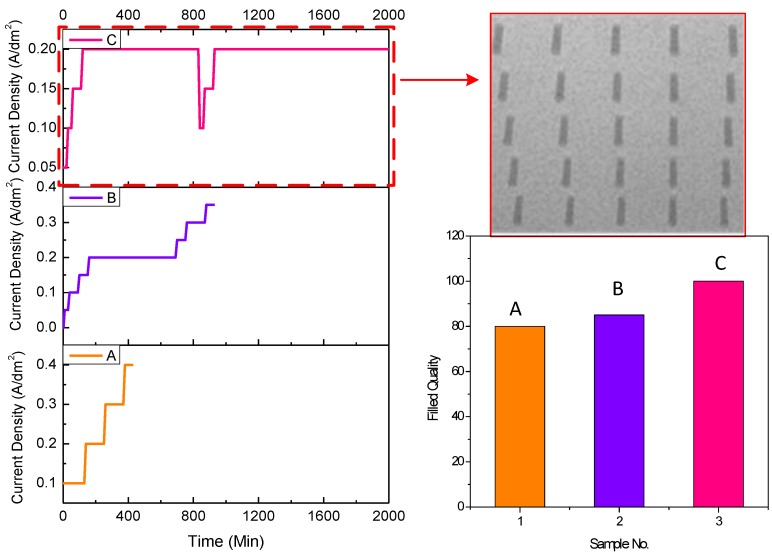
Illustration of the electroplating conditions and the filling quality of the samples 40 µm in diameter and 160 µm in depth. **A**, **B**, **C** are different electroplating conditions with various current densities and time shown in the figure.

**Figure 7:**
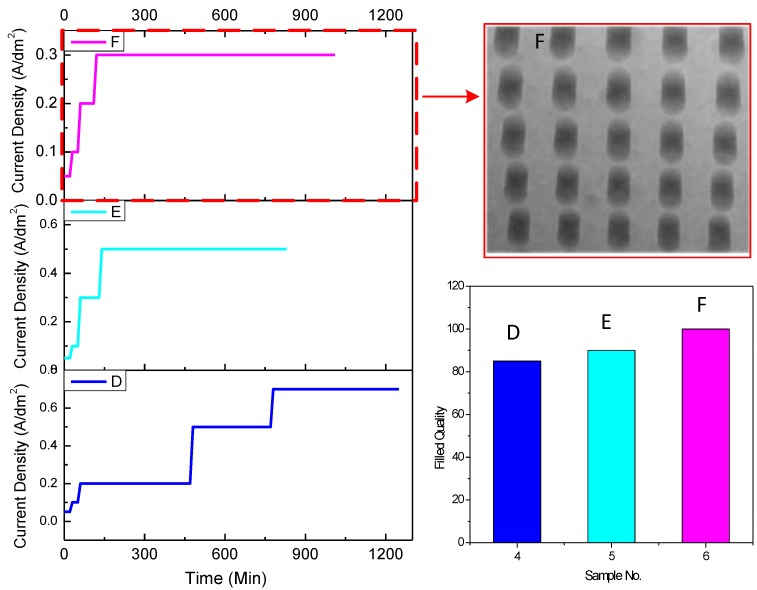
Illustration of the electroplating conditions and the filling quality of the samples 150 µm in diameter and 135 µm in depth. **D**, **E**, **F** are different electroplating conditions with various current densities and time shown in the figure.

The electroplating conditions and the filling quality of the samples 40 µm in diameter and 160 µm in depth are shown in Figure 6, while the conditions and the quality of samples 150 µm in diameter and 130 µm in depth are shown in Figure 7. For vias 40 µm in diameter and 160 µm in depth (Figure 6A), the starting current density was 0.1 A/dm^2^ and increased to 0.2, 0.3, and 0.4 A/dm^2^, in sequence. The maintainance times for the four parts of current density were 70, 80, 120, and 60 min, respectively. The results showed that almost 80% of the vias were filled. In order to improve the quality, lower starting current density and longer time were utilized, shown in Figure 6B,C. When the starting current density had decreased to 0.05 A/dm^2^, and the end current density was set to no more than 0.2 A/dm^2^, the vias were 100% filled, shown in the image inserted (Figure 6). For the vias 150 µm in diameter and 130 µm in depth, the starting current density was uniformly set to 0.05 A/dm^2^, and the stepwise current densities were varied, as shown in Figure 7(D–F). The stepwise current densities of 0.05 A/dm^2^ for 20 min, 0.1 A/dm^2^ for 30 min, 0.2 A/dm^2^ for 60 min, and 0.3 A/dm^2^ for 900 min (Figure 7F) performed the best. The figures demonstrate the distribution of the TSVs under different electroplating conditions. It can be seen that current density could be set to no more than 0.2 A/dm^2^ with several steps for vias 40 µm in diameter and 160 µm in depth (Figure 6), while the current density could be raised to 0.3 A/dm^2^ for vias 150 µm in diameter and 135 µm in depth (Figure 7).

It can be seen that the electroplating conditions including starting current density, stepwise electroplating set, and the end current density all had an effect on the filling quality. If the starting current density was too high, the opening would have been blocked quickly and influenced the electroplating. A stepwise current density with lower end current density weakened the opening blockage and increased the efficiency of the process. The mechanism of improvement can be explained that the pinch-off phenomenon was prevented by reducing the current accumulation effect around the opening when decreasing and introducing stepwise current density. Also, the stirring force during the electroplating could eliminate the restriction of the solution transportation.

**Figure 8:**
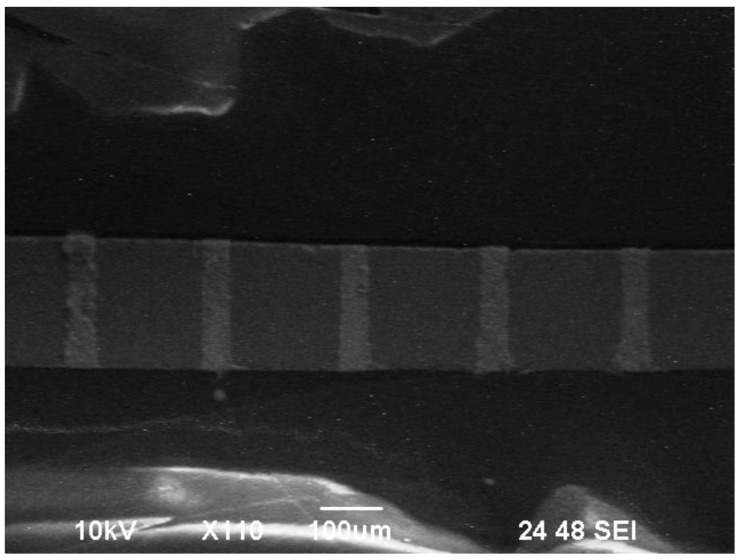
Scanning electron microscope (SEM) image of TSV filling quality.

The effect of the improvement in sputtering and electroplating condition was further proven by a sample 40 µm in diameter and 160 µm in depth. The SEM image shown in Figure 8 illustrates the perfect filling quality of the TSV.

## 4. Conclusions

In this paper, two approaches of improvement were carried out to fully fill Cu into the TSVs by electroplating. The effects of sputtering and electroplating conditions on the filling behavior of TSVs were analyzed. Based on the results of the experiments, it can be concluded that, according to the mechanism, filling occurs much easier on a Cu surface than a Ti surface. Since the filling condition can be controlled, a good filling quality without voids can be obtained. In addition, the dependence of the TSV electroplating condition was proven to have an effect on the improvement of the filling quality by setting stepwise current densities. A lower current density with longer time can fabricate better TSV filling due to its prevention of the pinch-off phenomenon.
